# Adherence to the Planetary Health Diet Index and correlation with nutrients of public health concern: an analysis of NHANES 2003–2018

**DOI:** 10.1016/j.ajcnut.2023.10.018

**Published:** 2024-01-05

**Authors:** Sarah M. Frank, Lindsay M. Jaacks, Linda S. Adair, Christy L. Avery, Katie Meyer, Donald Rose, Lindsey Smith Taillie

**Affiliations:** 1 Global Academy of Agriculture and Food Security, Royal (Dick) School of Veterinary Studies, The University of Edinburgh, Roslin, United Kingdom; 2 Carolina Population Center, University of North Carolina at Chapel Hill, Chapel Hill, NC, United States; 3 Department of Nutrition, Gillings School of Global Public Health, University of North Carolina at Chapel Hill, Chapel Hill, NC, United States; 4 Department of Epidemiology, Gillings School of Global Public Health, University of North Carolina at Chapel Hill, Chapel Hill, NC, United States; 5 Nutrition Research Institute, University of North Carolina at Chapel Hill, Kannapolis, NC, United States; 6 Tulane Nutrition, School of Public Health and Tropical Medicine, Tulane University, New Orleans, LA, United States

**Keywords:** EAT-Lancet Commission, dietary patterns, trends, Planetary Health Diet, nutrients of public health concern, NHANES

## Abstract

**Background:**

The Planetary Health Diet Index (PHDI) is a novel measure adapted to quantify alignment with the dietary evidence presented by the EAT-*Lancet* Commission on Food, Planet, Health.

**Objectives:**

To examine how population-level health and sustainability of diet as measured by the PHDI changed from 2003 to 2018, and to assess how PHDI correlated with inadequacy for nutrients of public health concern (iron, calcium, potassium, and fiber) in the United States.

**Methods:**

We estimated survey-weighted trends in PHDI scores and median intake of PHDI components in a nationally representative sample of 33,859 adults aged 20+ y from 8 cycles (2003–2018) of the National Health and Nutrition Examination Survey with 2 d of dietary recall data. We used the National Cancer Institute method to examine how PHDI correlated with inadequate intake of iron, calcium, potassium, and fiber.

**Results:**

Out of a theoretical range of 0–140, the median PHDI value increased by 4.2 points over the study period, from 62.7 (95% confidence interval [CI]: 62.0, 63.4) points in 2003–2004 to 66.9 (66.2, 67.7) points in 2017–2018 (*P*-trend < 0.001), although most of this change occurred before 2011–2012 and plateaued thereafter. For adequacy components that are encouraged for consumption, nonstarchy vegetable intake significantly decreased over time, whereas whole grains, nuts and seeds, and unsaturated oils increased. For moderation components with recommended limits for consumption, poultry and egg intake increased, but red and processed meat, added sugars, saturated fats, and starchy vegetables decreased over time. Higher PHDI values were associated with a lower probability of iron, fiber, and potassium inadequacy.

**Conclusions:**

Although there have been positive changes over the past 20 y, there is substantial room for improving the health and sustainability of the United States diet. Shifting diets toward EAT-*Lancet* recommendations would improve nutrient adequacy for iron, fiber, and potassium. Policy action is needed to support healthier, more sustainable diets in the United States and globally.

## Introduction

Diet, climate change, and human health are closely interrelated. Global dietary shifts are associated not only with increased risk of obesity, type II diabetes, cardiovascular disease, and certain cancers [[1], [2], [3], [4], [5], [6], [7]], but also with intensive production methods that contribute to environmental degradation via greenhouse gas emissions, land use change, land degradation, and water pollution [4,[8], [9], [10]]. A 2021 report from the Intergovernmental Panel on Climate Change warned that climate change and its effects on human health are accelerating, and there is a dire need for solutions across a variety of sectors, including the food system [11,12].

To better align nutrition and sustainability targets, in 2019, the EAT-*Lancet* Commission on Food, Planet, Health introduced the “universal healthy reference diet,” also referred to as the Planetary Health Diet (PHD) [1] to reduce the burden of diet-related disease and minimize the environmental impact of feeding human populations. The reference diet provides 2500 kcal/d and adequate macro- and micronutrient intakes and was evaluated against planetary boundaries for 6 key environmental indicators.

In the United States, components of the EAT-*Lancet* universal healthy reference diet have been compared with components of the Healthy Eating Index—which measures adherence to the Dietary Guidelines for Americans (DGAs)—but only for individual food groups rather than comparing the 2 dietary patterns overall [13]. To the best of our knowledge, no study has applied a diet index based on evidence from the EAT-*Lancet* Commission to describe the health and sustainability of diets in a nationally representative United States population, nor how adherence to such a diet has changed in recent years, because awareness of the environmental impact of diet has grown [14].

The EAT-*Lancet* Commission recommends a dietary pattern high in plant-based foods, including protein foods, and low in animal-sourced products, such as meat, fish, and dairy [1]. The typical United States diet, on the other hand, is characterized by a high intake of animal-sourced foods and a low intake of beans, legumes, and other iron-rich plant sources [15]. Indeed, meat and poultry are the top food sources of dietary iron in the United States [16,17].

Because the Planetary Health Diet Index (PHDI) is a novel dietary measure, and because it has several key differences from the DGAs [13], we tested the correlation of PHDI with the adequacy of key micronutrients of public health concern in the United States. We decided to evaluate iron because animal-sourced foods are a major source of dietary iron and calcium in the typical American diet [16,17], whereas the PHDI recommends a low intake of most animal-sourced foods. Other micronutrients of concern that are lacking in many American diets are calcium, potassium, fiber, and vitamin D [18]. In nationally representative data, the prevalence of inadequacy among United States adults was estimated to be 95% for fiber [19], 70% for potassium, and 44% for calcium [20]. Although a shift toward the EAT-*Lancet* universal healthy reference diet would likely improve intakes of fiber and potassium given that vegetables, beans, legumes, and fruit are rich sources of these micronutrients, the impact on calcium and iron intakes is uncertain and evidence on the recommendation’s correlation with nutrient intake in real-world settings is limited.

The objectives of this study were to assess how the United States diet aligns with the PHDI, a novel index based on the evidence presented by the EAT-*Lancet* Commission, and to examine changes in accordance with the PHDI between 2003 and 2018 for the entire dietary pattern and its constituent components. We further assess how PHDI correlates with inadequacy for key nutrients of public health concern in the United States (iron, fiber, potassium, and calcium).

## Methods

### Study population

The United States NHANES is a repeated cross-sectional survey that uses a multistage probability design to sample the civilian, noninstitutionalized population residing in the 50 states and the District of Columbia [21]. The survey was approved by the Ethics Review Board of National Center for Health Statistics, and all participants provided written informed consent [21]. Because the deidentified observational data are publicly available for download, this study received a determination of Not Human Subjects Research by the Institutional Review Board at the University of North Carolina at Chapel Hill.

Eligible participants were nonpregnant or lactating individuals aged ≥20 y who participated in any cycle of NHANES from 2003 to 2018 (8 cycles in total) and for whom 2 d of valid dietary intake data were available (Supplemental Figure 1). Participants whose mean intake was <500 kcal/d or >8000 kcal/d were excluded [22] (*N* = 147).

### Dietary data

Trained interviewers used the USDA Automated Multiple Pass Method to gather 24-h dietary recall data [23]. Participants were asked to recall all foods and beverages they consumed the previous day. Measuring guides were used to assist with approximating the portion sizes of consumed foods. The second dietary interview was conducted unannounced via telephone 3–10 d after the initial face-to-face interview.

Dietary recall data were merged into the Food Patterns Equivalent Database (FPED), which assigns foods to the 37 USDA Food Pattern Components using a food composition table. For single-ingredient food items, FPED assigns foods directly to the corresponding component. For multi-ingredient foods with ingredients from >1 component, FPED disaggregates these items into their component ingredients’ gram weights using standard recipe files [24].

Thirty-five FPED components are published in nongram units (e.g., cup-equivalents, ounce-equivalents, teaspoon-equivalents, etc.) into grams. We used data from the Food Patterns Ingredients Database to assign the gram weights required for score derivation to these 35 FPED components by merging FPED into Food Patterns Ingredients Database. Multi-ingredient dishes were broken down into their constituent ingredients by proportional contribution of weight (see Supplemental Table 1 for an example of our approach and link to Python script). After the data were flattened and all FPED components were available in grams, the mean of 2-d intake, in grams, was calculated for each component. Because cow milk is ∼90% water, producing equivalent weights of dairy products, such as cheese takes more milk and changes the proportion of milk solids and nutrient content in a given product [24]. To better represent the nutrient density and environmental impact of the various dairy foods (e.g., milk compared with cheese) dairy servings are often represented as “whole-milk or derivative equivalent” [1,[24], [25], [26]]. We used FPED’s cup-equivalents of dairy to define a serving-equivalent of dairy. This reflects the use of whole-milk or derivative equivalents without misrepresenting the actual number of grams reported by participants.

Total energy intake (TEI) was derived from the mean of 2 d of total intake reported on the dietary questionnaires and included in all models to control for confounding and reduce extraneous variation in dietary variables [27].

### Derivation of the PHDI

The PHDI was derived from the self-reported intake of 14 food groups in accordance with the midpoint of the recommended range listed in the EAT-*Lancet* Commission Scientific Report and validated by Bui et al. [1,28]. To be consistent with the EAT-*Lancet* report [1], grams were used as the primary unit of measurement for each food group rather than calories. The exception for grams was dairy foods, for which we converted the EAT-*Lancet* recommendations of grams to serving-equivalents based on the FPED conversion of one cup whole-milk equivalent = 245 g [24] (see Dietary data and Table 1).

**TABLE 1:** Scoring criteria for the Planetary Health Diet Index

Dietary component	Category minimum score (0 points)	Category maximum score (10 points)
Adequacy components		
Whole grains^1^	0 g	≥75 g for women
		≥90 g for men
Whole fruits (excludes fruit juice)	0 g	≥200 g
Nonstarchy vegetables	0 g	≥300 g
Nuts and seeds	0 g	≥50 g
Legumes		
Nonsoy legumes^2^^,^^3^	0 g	100 g
Soybean/ soy foods^2^^,^^3^	0 g	50 g
Unsaturated oils	0% of total energy intake	≥10% of total energy intake
Moderation components		
Starchy vegetables	≥200 g	≤50 g
Dairy^4^	≥4.08 serving-equivalents	≤1.02 serving-equivalents
Red and processed meat	≥300 g	≤14 g
Poultry	≥58 g	≤29 g
Eggs	≥120 g	≤12 g
Fish	≥50 g	≤15 g
Saturated oils and *trans* fats	≥21% of total energy intake	≤3.5% of total energy intake
Added sugar and fruit juice	≥25% of total energy intake	≤5% of total energy intake

1 Thresholds were based on the midpoint of the recommended range listed in EAT-*Lancet* Commission Scientific Report [1].

2 Grams per day calculated from dry weight.

3 To calculate the score for the legumes component, the nonsoy and soy subcomponents were each weighted at 0.5.

4 In FPED, 1 serving of dairy is equal to 245 g of whole-milk or derivative equivalent. In the EAT-*Lancet* report, scores were assigned ≤250 g whole-milk or derivative equivalent for the maximum score or ≥1000 g whole-milk or derivative equivalent for the minimum score.

For each food group, participants received a score ranging from 0 (minimum) to 10 (maximum) (Table 1). Intakes between the minimum and the maximum levels were scored proportionately, as others have used for scoring of dietary indices [29].

This coding is distinct from previous weight-based calculations of the EAT-*Lancet* Commission’s reference diet in that it uses continuous rather than binary scoring to allocate points [30,31], resulting in a wider range of participant scores to better capture population-level variability in diet. For the moderation components, the use of evidence-based minimum and maximum thresholds [28] with proportional scoring in between better represents dietary risk than the assignment of binary scores – e.g., having an intake of added sugars slightly above the recommended amount has different implications than consuming at levels well above the recommendation. Finally, for consistency with the EAT-*Lancet* report and to be more conservative, we used midpoints estimates from the Commission’s healthy reference diet (as done for other dietary indices [32]) rather than an end point of the possible range [[29], [30], [31]].

Of the 14 food groups, 6 (whole grains, whole fruits, nonstarchy vegetables, nuts and seeds, legumes, and unsaturated oils) were Adequacy components, and were encouraged for consumption such that intakes at or above the maximum threshold were scored at the maximum 10 points. As recommended by the Commission Report, legumes were divided into 2 subgroups—nonsoy legumes and soybean/soy foods—each of which was weighted at 0.5 for the purpose of score derivation [1]. The remaining 8 food groups (starchy vegetables, dairy, red and processed meat, poultry, eggs, fish, saturated oils and *trans* fats, added sugar and fruit juice) were Moderation components and were generally discouraged from consumption, in which intakes at or approaching 0 were awarded the maximum 10 points.

Once the component scores were assigned, the scores for all 14 components were summed to create a total score. Therefore, the maximum possible score for the PHDI was 140.

### Micronutrients of concern

For all micronutrients of concern, intake from food was available in milligrams per day (mg/d).

Although vitamin D is also considered a nutrient of concern for the United States population, we did not include analyses of vitamin D because data on vitamin D intake from food were not available for the 2003–2004 and 2005–2006 NHANES cycles.

### Sociodemographic variables

All sociodemographic information was self-reported as part of a standardized questionnaire. Age data were modeled in continuous years. Income data were classified using the Poverty Income Ratio (PIR), a measure of family income relative to the Federal Poverty Level that accounts for household size. Income was categorized as PIR 0%–185%; PIR 186%–399%; PIR ≥400; and Missing (because of high missingness in self-reported income, 6.3%) [33]. Education was reported in continuous years and classified as high school equivalent or lower; some college; and college degree or higher [34]. Race/ethnicity data were self-reported via categorical selection and classified as following: *1*) Non-Hispanic White; *2*) Non-Hispanic Black; *3*) Hispanic; and *4*) Non-Hispanic Asian, or Other race/ethnicity (including Multiracial) [33,35].

### Statistical analyses

To assess differences in PHDI score and for PHDI components across the years, we modeled survey years as binary variables in survey-weighted quantile regression. To assess overall trends over the entire study period (2003–2018), *P*-trend was calculated by modeling survey year as a continuous variable in survey-weighted quantile regression. Models were adjusted for TEI. For the descriptive analysis of disparities in PHDI score, chi-square statistics were used to test for demographic differences. All descriptive analyses were conducted in Stata version 17.0.

For calcium, potassium, and fiber, we calculated the prevalence of inadequacy from food intake—that is, without the use of dietary supplements—using the Simulating Intake of Micronutrients for Policy Learning and Engagement (SIMPLE) macro, which is an implementation of the National Cancer Institute’s method for calculating usual intake from 24-h recall data [36]. We used the standard SIMPLE macro for calcium, potassium, and fiber, which are normally distributed. Because the distribution of iron adequacy is skewed, we used the SIMPLE-Iron macro, a variation of the SIMPLE macro that uses a full probability method, to calculate iron inadequacy [36,37]. Age, sex, income, educational attainment, race/ethnicity, and TEI were all included as covariates to improve precision in the estimation of the usual intake of nutrients [38]. Analyses of nutrient adequacy were conducted in SAS v9.4. *P* value of <0.05 was considered statistically significant for all analyses.

## Results

The final analytic sample included 33,859 participants. PHDI scores ranged from a minimum of 18.5 to a maximum of 125 out of a theoretical range of 0–140 [median = 66.0 (interquartile range: 57.0, 75.0), Table 2]. Across the 15-y time period, the prevalence of iron inadequacy was low (4.1%), whereas 43.5% of the population had inadequate calcium intake, 67.0% had inadequate potassium intake, and 92.3% had inadequate fiber intake.

**TABLE 3:** Distribution of population characteristics by quintile of PHDI, NHANES 2003–2018^1^

	PHDI quintile					*P* value^2^
	1	2	3	4	5	
Diet quality score (range)	18.5–54.0	54.5–62.0	62.5–69.0	69.5–77.0	77.5–125.0	
Sex						<0.001
Male	24.7 (23.6, 25.9)	22.7 (21.8, 23.7)	18.3 (17.5, 19.2)	17.1 (16.2, 18.1)	17.1 (16.0, 18.2)	
Female	14.6 (13.6, 15.6)	19.4 (18.4, 20.3)	20.9 (20.1, 21.8)	21.4 (20.4, 22.4)	23.8 (22.4, 25.2)	
Age, mean (95% CI) (y)	43.1 (42.6, 43.7)	45.7 (45.1, 46.4)	48.5 (47.8, 49.2)	50.1 (49.3, 50.9)	51.3 (50.5, 52.0)	<0.001
Education						<0.001
High school equivalent or lower	24.4 (23.2, 25.7)	23.9 (22.8, 25.0)	20.9 (19.9, 21.9)	16.9 (16.1, 17.8)	14.0 (13.0, 15.0)	
Some college	21.1 (19.8, 22.6)	22.1 (20.8, 23.5)	19.0 (17.8, 20.2)	19.2 (18.1, 20.3)	18.6 (17.2, 20.1)	
College degree or greater	11.3 (10.1, 12.5)	16.0 (14.7, 17.4)	18.8 (17.5, 20.1)	22.7 (21.1, 24.3)	31.3 (29.4, 33.2)	
Income						<0.001
Poverty-to-income ratio <185%	24.9 (23.6, 26.2)	23.2 (22.1, 24.4)	20.0 (19.1, 21.0)	17.3 (16.4, 18.2)	14.6 (13.5, 15.8)	
Poverty-to-income ratio 185%–399%	21.1 (19.8, 22.6)	20.9 (19.7, 22.3)	19.3 (18.1, 20.4)	19.3 (17.9, 20.8)	19.4 (18.1, 20.9)	
Poverty-to-income ratio ≥400%	13.8 (12.6, 15.1)	19.0 (17.7, 20.3)	20.0 (18.7, 21.5)	20.8 (19.5, 22.1)	26.5 (24.9, 28.1)	
Missing income information	18.2 (15.8, 20.9)	22.1 (19.5, 25.0)	18.0 (15.8, 20.4)	21.2 (18.7, 23.9)	20.5 (17.7, 23.6)	
Race/ethnicity						<0.001
Non-Hispanic White	17.5 (16.4, 18.6)	20.6 (19.7, 21.6)	19.9 (19.1, 20.8)	20.1 (19.2, 21.1)	21.8 (20.5, 23.2)	
Non-Hispanic Black	32.6 (30.8, 34.5)	24.3 (22.9, 25.8)	18.3 (17.1, 19.4)	14.5 (13.4, 15.6)	10.3 (9.2, 11.5)	
Hispanic	20.9 (19.7, 22.1)	22.5 (21.0, 24.0)	20.4 (19.0, 21.7)	19.2 (18.0, 20.5)	17.1 (15.7, 18.6)	
Asian, Multiracial, and Other Non-Hispanic race/ethnicities	16.1 (14.1, 18.3)	16.5 (14.7, 18.6)	18.0 (16.1, 20.2)	19.2 (17.2, 21.3)	30.2 (27.5, 32.9)	

Abbreviation: PHDI, Planetary Health Diet Index.

1 All values are survey-weighted proportion (95% confidence interval) unless otherwise noted.

2 *P* values are from chi-square tests for the effect at the overall demographic level.

**FIGURE 1:**
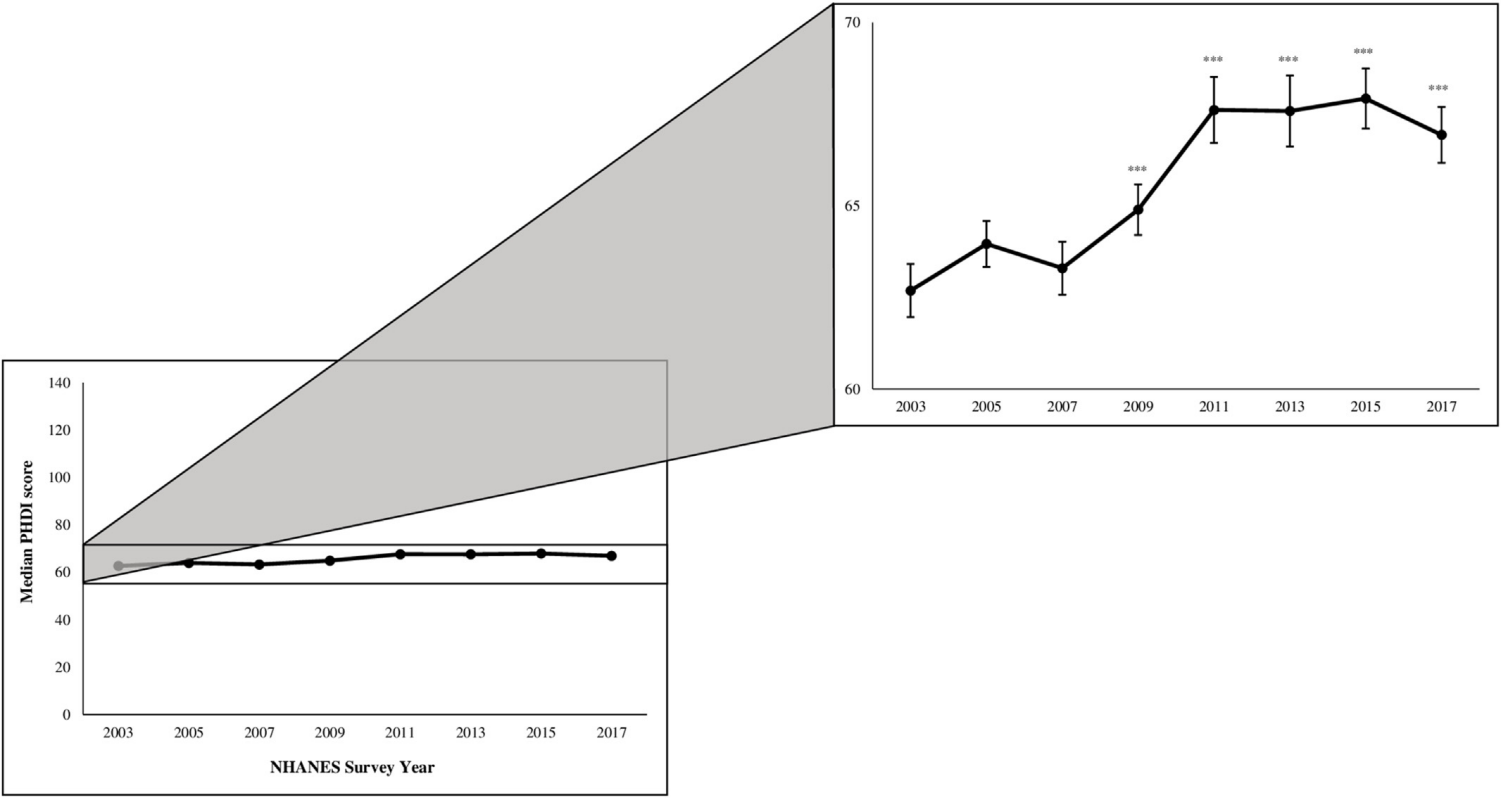
Changes in median Planetary Health Diet Index score, NHANES 2003–2018.1,2 ^1^Quantile regression model was adjusted for total energy intake. ^2^∗*P* < 0.05, ∗∗*P* < 0.01, and ∗∗∗*P* < 0.001 for the difference from the 2003–2004 NHANES cycle.

Overall, the PHDI score improved over time (Figure 1). The estimated increase in median PHDI score was 0.38 (95% CI: 0.31, 0.44) points per survey cycle, with a predicted median PHDI of 62.7 (62.0, 63.4) in 2003–2004, compared with 66.9 (66.2, 67.7) in 2017–2018 (*P*-trend < 0.001). However, the median PHDI in 2011–2012 [67.6 (66.7, 68.5)] did not differ significantly from the median PHDI in 2017–2018. We also compared changes in median intake for the lowest and highest quintiles of PHDI score over time. The median PHDI score in quintile 1 increased by 4.2 points, from an estimated 47.3 (95% CI: 46.6, 48.1) in 2003–2004 to 51.5 (50.4, 52.6, *P* < 0.001) in 2017–2018. For quintile 5, the median PHDI increased by 6.8 points, from an estimated 78.7 (77.7, 79.8) in 2003–2004 to 85.5 (84.2, 86.8) in 2017–2018. There were no significant changes in median PHDI between 2011–2012 and 2017–2018 for either quintile 1 or quintile 5 (Supplemental Table 2).

In addition, we estimated the median intake of the PHDI components and changes in these components over time (Supplemental Table 3). Median intake of all adequacy components except added fat—unsaturated oils was below PHDI recommendations. Consumption of nonstarchy vegetables significantly decreased over time [136.2 g (130.1, 142.2) in 2003–2004 compared with 118.7 g (111.9, 125.4) in 2017–2018, *P* < 0.001]. However, there were modest but significant increases in whole grains [16.0 g (13.6, 18.4) compared with 23.9 g (20.2, 27.6), *P* < 0.001], nuts and seeds [1.3 g (1.0, 1.5) compared with 2.2 g (1.5, 3.0), *P* < 0.01], and added fat—unsaturated oils [6.1% of TEI (5.9, 6.3) compared with 10.3% of TEI (10.0, 10.6)]. There were no statistically significant changes in the consumption of soy, nonsoy legumes, or fruit.

For the moderation components, the median intake of starchy vegetables, poultry, and eggs aligned with PHDI recommendations, whereas the intake of red and processed meat and added fat—saturated oils and *trans* fat were above PHDI recommendations (Supplemental Table 3). Consumption of starchy vegetables [47.8 g (44.4, 51.2) in 2003–2014 compared with 39.0 g (35.0, 43.0) in 2017–2018, *P* < 0.001] added fat—saturated oils and *trans* fat [9.8% of TEI (9.5, 10.1) compared with 7.5% of TEI (7.2, 7.8, *P* < 0.001)], and added sugar and fruit juice [14.9% of TEI (14.4, 15.4) compared with 11.9% of TEI (11.4, 12.4), *P* < 0.001] significantly decreased over time. In addition, consumption of poultry [23.1 g (19.3, 26.7) compared with 30.5 g (26.7, 34.3), *P* < 0.01] and eggs [8.6 g (7.6, 9.7) compared with 13.1 g (11.3, 15.0), *P* < 0.001] significantly increased. There were no statistically significant changes in consumption of dairy, fish, or red and processed meat.

We observed several disparities in diet quality as measured by PHDI (Table 3). A higher proportion of males were in the lowest PHDI quintile as opposed to the highest quintile, whereas the opposite was true for females. Individuals in the highest income category, with a college degree or greater, and who self-identified as Non-Hispanic White or Asian, Multiracial, and Other Non-Hispanic ethnicity were more likely to be in the highest PHDI quintile. Conversely, individuals with the lowest level of income and education, as well as those who self-identified as Non-Hispanic Black or Hispanic, were more likely to be in the lowest quintile of PHDI score.

**TABLE 2:** Characteristics of eligible participants with 2 d of dietary recall data, NHANES 2003–2018^1^

Sex	
Male	48.8 (16,611)
Female	51.2 (17,248)
Mean (SD) age (y)	47.8 (17.0)
Educational attainment	
High school equivalent or lower	39.0 (15,977)
Some college	31.6 (10,027)
College degree or greater	29.4 (7822)
Income	
Poverty-to-income ratio <185%	29.7 (13,593)
Poverty-to-income ratio 185%–399%	29.4 (9413)
Poverty-to-income ratio ≥400%	34.6 (8223)
Missing income information	6.3 (2630)
Race/ethnicity	
Non-Hispanic White	68.3 (15,370)
Non-Hispanic Black	11.3 (7253)
Hispanic	13.3 (8115)
Asian, Multiracial, and Other Non-Hispanic race/ethnicities	7.1 (3121)
Median (IQR) energy intake (kcal/d)	1969 (1523–2542)
Median (IQR) Planetary Health Diet Index values	66.0 (57, 75)
Inadequate iron intake^2^ (%)	4.1 (3.8, 4.3)
Inadequate calcium intake^2^ (%)	43.5 (42.2, 44.8)
Inadequate potassium intake^2^ (%)	67.0 (65.7, 68.4)
Inadequate fiber intake^2^ (%)	92.3 (91.5, 93.1)

Abbreviation: IQR = Interquartile Range.

1 Values are weighted % (unweighted *N*) unless otherwise noted. Weighted % accounts for complex survey weights.

2 Results are from the Simulating Intake of Micronutrients for Policy Learning and Engagement macro wrapper of the National Cancer Institute Method for estimating usual intake and were adjusted for age, sex, income, education, race/ethnicity, and total energy intake.

**FIGURE 2:**
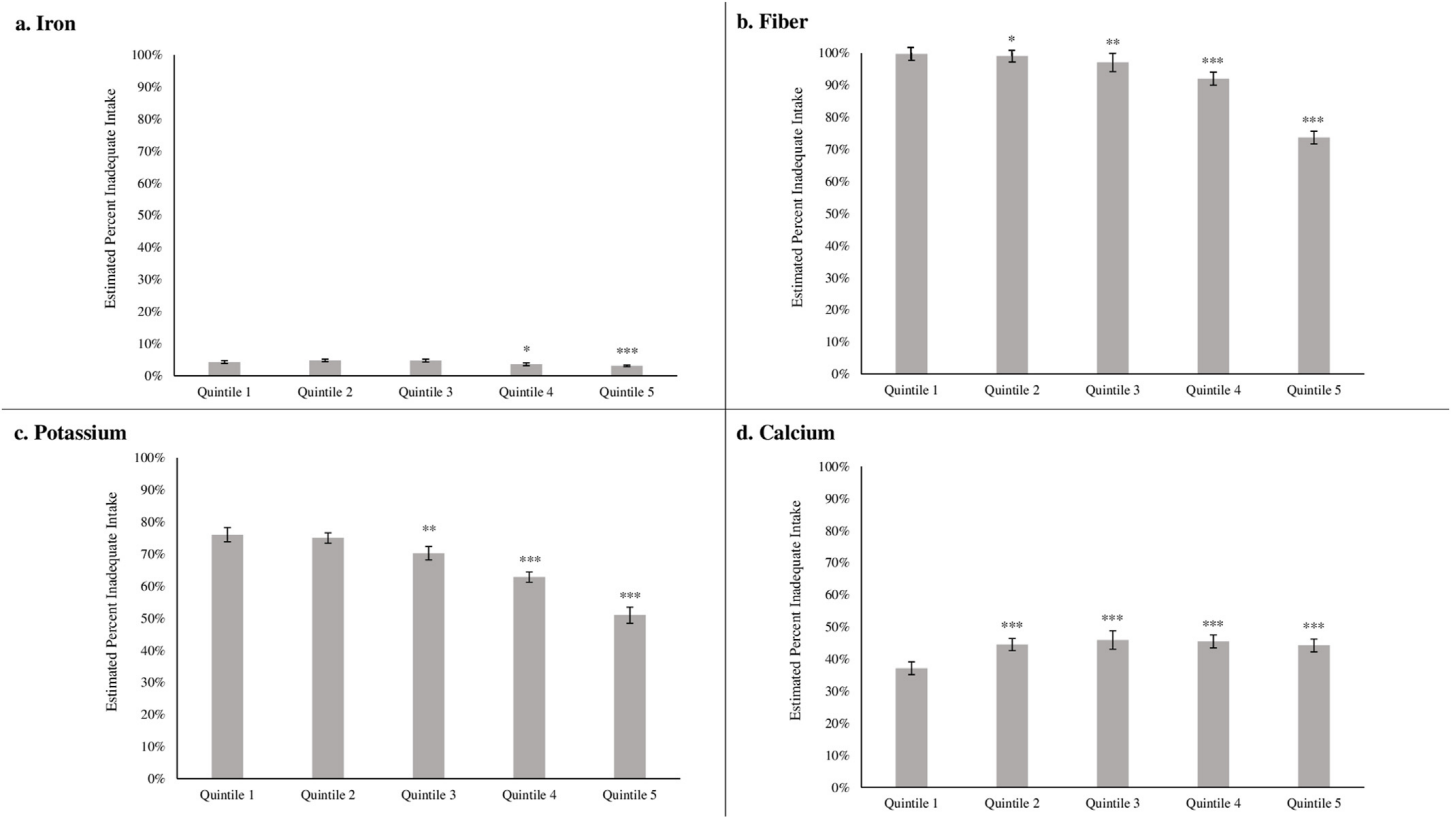
Predicted probability of meeting the recommended daily allowance for iron by quintile of Planetary Health Diet Index score, NHANES 2003–2018.1,2 ^1^Quantile regression models were adjusted for total energy intake.^2^∗*P* < 0.05, ∗∗*P* < 0.01, and ∗∗∗*P <* 0.001 for the difference from quintile 1.

Finally, we assessed the correlation of the PHDI quintile with key nutrients of concern for the American population. We observed an inverse association between PHDI quintile and probability of inadequate iron intake: 4.3% (3.8, 4.7) of those in quintile 1 had inadequate iron intake, compared with 3.1% (2.8, 3.3) of those in quintile 5 (*P*-trend < 0.01, Figure 2A, Supplemental Table 4). For fiber intake, 99.8% (99.7, 99.9) of those in quintile 1 had inadequate fiber intake, compared with 73.7% (71.4, 76.0) of those in quintile 5 (*P*-trend < 0.001, Figure 2B). Similarly, the predicted probability of inadequate potassium was higher for quintile 1 [76.1% (73.8, 78.3)] than for those in the quintile 5 [51.0% (48.5, 53.5), *P*-trend < 0.001, Figure 2C]. On the other hand, the predicted probability of inadequate calcium intake was lower in PHDI quintile 1 [37.1% (35.1, 39.2)] than any other PHDI quintile [e.g., 44.3% (42.3, 46.3) for quintile 5, *P* < 0.001, Figure 2D].

## Discussion

The typical American diet—as indicated by our results—is still far from aligning with the evidence presented by the EAT-*Lancet* Commission on Food, Planet, and Health. In the 2017–2018 survey cycle, the median PHDI score was 66.9, less than half of the theoretical maximum score of 140 and only 4.2 points greater than in 2003–2004. Notably, many of the improvements occurred during the middle of the time period. Consistent with findings that United States dietary quality improved in the mid-2000s (2005–2011) and then plateaued [39], we similarly find that improvements in PHDI score have stalled since the early 2010s. We also find disparities by income, education, and race/ethnicity consistent with well-established evidence on dietary disparities in the United States [40]. Current policies have not done enough to promote healthy eating, and urgent policy action is needed to improve the nutritional quality and sustainability of United States diets.

The low median PHDI scores and relative lack of progress observed here are driven by several underlying components. For moderation components, the United States is above targets for added sugars, added fat—saturated oils and *trans* fats, dairy, and red and processed meat, reflecting the typical “Western-style” dietary pattern. The United States diet is particularly high in terms of red and processed meat intake, with a median value of 65.9 g/d approximately 5 times the 14 g/d proposed by the EAT*-Lancet* Commission. Moreover, we observed no change in dairy or red and processed meat intake, coupled with an increase in poultry and eggs. This is consistent with other findings of animal-sourced food intake in the United States [41].

At the same time, we observed an inverse association between PHDI score and iron inadequacy. Such a pattern has been observed elsewhere [42] and mitigates some concerns that the PHD might be linked to poorer iron status because of lower meat intake in high-income settings. Instead, high intake of meat is associated with cardiovascular disease, type II diabetes, and certain cancers, and production of meat and dairy has significant impacts on greenhouse gas emissions, water use, land use, and biodiversity loss [43]. In this context, our findings of high overall animal-sourced food intake coupled with the inverse association between PHDI and iron inadequacy suggest that public health and environmental outcomes in the United States could be improved by reducing animal-sourced foods without increasing the burden of anemia.

In addition to overconsumption of moderation components, we found under consumption for several adequacy components, namely whole grains, fruits, vegetables, legumes, and nuts and seeds. Similar to other studies in NHANES that found whole grain and nuts and seeds intake to be low but steadily increasing since the turn of the 20th century [44], we observed small but significant increases in consumption of these food groups and intake levels well below recommended amounts. We also observed decreases in fruit and nonstarchy vegetable intake over the study period. Adherence to fruit and vegetable recommendations in the United States has been and remains low [45], and there is evidence of a decreasing probability of fruit intake among United States adults in recent years [46]. Insufficient intakes of adequacy components—particularly for whole grains—are leading causes of morbidity and mortality in the United States [32], and our study further highlights the need for ambitious public health efforts to improve intakes of these foods.

Corresponding to the low intakes of whole grains, nuts and seeds, fruits, and vegetables across the population, we also found low intakes of fiber and potassium. However, those with higher PHDI scores were less likely to have inadequate intakes of fiber and potassium, corresponding to relatively higher intakes of these foods. Although those with higher PHDI scores also had slightly higher calcium inadequacy, many vegetables, seeds, and legumes have a higher density [47] and bioavailability [48] of calcium than dairy products. Given the unclear relationship between dairy and health [1,49] and the environmental impacts of dairy production, promoting a greater intake of plant-based foods rich in calcium, such as leafy greens, seeds, edamame, and tofu could improve calcium adequacy as well as fiber and potassium adequacy. Overall, healthy plant-based diets rich in the adequacy components are associated with better nutrient intakes, health, and environmental outcomes [50] and increasing the intake of these foods is crucial to improve the health and sustainability of United States diets.

Indeed, we found that not only are United States diets unhealthy and unsustainable, but that there have not been meaningful improvements in dietary quality in the 21st century. There are several factors that contribute to the persistence of unhealthy diets in the United States. The first is the influence of multinational food corporations, which have become increasingly concentrated and thus increasingly powerful actors with considerable control over the food supply and significant political influence [51]. For example, corporate interests have directly impacted United States dietary policy via continued involvement in the DGAs [52,53]. Lobbying for subsidies keeps the price of a select few commodities, such as red meat, dairy, and corn (often used in ultraprocessed foods) artificially low and floods the market with these products without truly accounting for their health or environmental costs [51,54]. In addition, stagnant wages coupled with an increasing cost of food makes low-cost ultra-processed foods (UPFs) that are often high in moderation components (added sugars, saturated fats, etc.) attractive to busy households trying to make ends meet [55]. UPFs are largely discretionary foods but make up over half of the average American’s calories [56] and involve intensive packaging, processing, and transportation. Because UPFs account for such a large part of the diet, many resources used in and impacts of our current food systems are for foods that are neither healthy nor sustainable [57]. At the same time, most subsidies do not directly cover tree nuts, fruits, or vegetables: <1% of federal crop subsidies go to specialty crops, resulting in <3% of domestic cropland being used for vegetables, orchards, and berries [58,59]. Simply put, the current political, economic, and social environment of the United States does not support a robust transition to healthier, more sustainable diets. Such a transformation will require public and political will, multisectoral cooperation and ambitious policies in food, economics, and agriculture.

Given the stalled progress toward improved dietary quality, there are several potential policy avenues to improve the health and sustainability of the United States diet. The process of drafting the 2025–2030 DGAs began in early 2023 and presents an opportunity for the United States to address the sustainability as well as the healthfulness of diet. Because the DGAs are the basis for all federal food programs, the subsequent dietary shifts would have significant benefits for health and environmental outcomes [60]. In addition, policies, such as redistributing agricultural subsidies to provide fewer subsidies for meat, dairy, corn, and soy and more for fruit and vegetable production, could alter the United States food system to promote healthier, more sustainable diets [61]. Disincentives, such as taxes or warning labels on red meat and added sugars, could also be leveraged. Affordability of food is a major barrier to consuming a healthy diet [62] and consumer subsidies for healthy foods increase purchases of fruits, vegetables, nuts, and legumes [63]. Policy efforts on multiple fronts are needed to promote the health and sustainability of diets in the United States.

Beyond the United States context, results from other studies have shown that better adherence to the PHD is correlated with higher nutrient intakes [29], lower risk of ischemic heart disease and type II diabetes, [30] lower incidence of cancer-, cardiovascular-, and all-cause mortality [42], and lower dietary emissions [25,29]. Of note, most of these studies have occurred in high-income countries in which undernutrition is not a major public health concern.

Beyond these high-income settings, several studies have suggested that the PHD may not provide adequate intake of certain nutrients, particularly for special populations, such as people who menstruate or who are pregnant [31,64]. The country or regional context and flexibility of the PHD matter from an ethical and equity perspective: for many nutritionally vulnerable populations, intake of animal-source foods is lower than the thresholds presented by the EAT-*Lancet* Commission and the majority of energy comes from starchy carbohydrates, making animal-source foods a valuable source of micronutrients. When thinking about global malnutrition, great care needs to be taken to ensure that after the PHD accounts for the burden of nutrient deficiencies in local contexts [1,64].

The PHDI tool presented here, although tested in a United States population, is designed for use in a variety of settings. Global diets are neither as healthy nor as sustainable as the EAT-*Lancet* Commission reference diet, but there is significant heterogeneity in how diets diverge from the recommendations. The PHDI can capture this heterogeneity and identify tailored areas for improvement. It can be used to set national food, diet, and agricultural priorities, particularly for countries that are in earlier stages of the nutrition transition. In addition, applying the PHDI in diverse global settings can provide a unified framework to directly compare the health and sustainability of diet across countries and track progress over time. It could be used in conjunction with tools, such as the Food Systems Dashboard [65], to inform global food systems governance and work toward healthier, more sustainable food systems for all.

The present study had several limitations. We used data from 24-h dietary recalls, which cannot capture usual intake for individuals. However, the use of NHANES survey weights allowed us to obtain nationally representative, population-level estimates for PHDI and component scores, and we used a validated methodology to estimate nutrient adequacy from 2 recalls [36]. In addition, we did not account for the use of supplements in our adequacy analyses. However, the goal of EAT-*Lancet* is to provide a diet that is nutritionally adequate without the need for supplements, and we assessed its performance for nutrient intake from food. The EAT-*Lancet* report published ranges of values for each component to allow for more flexibility [1,13], but for the simplicity of these analyses, we used the Report’s point estimates to calculate our score. Similarly, although we used the most recently available waves of NHANES data, we were unable to account for changes because of the COVID-19 pandemic. Future research should seek to quantify how adherence to the PHDI has changed during the pandemic and its aftermath.

In conclusion, to our knowledge, this paper is among the first to analyze adherence to the EAT-*Lancet* universal healthy reference diet in a nationally representative sample of United States adults. We find that although there have been small, positive changes over the past 20 y, there is substantial room for improving the health and sustainability of the United States diet. Shifting United States diets toward the EAT-*Lancet* recommendations would improve nutrient adequacy for iron, fiber, and potassium. Policy action is needed to transform food systems and accelerate the transition to healthier, more sustainable diets in the United States.

## Author contributions

The authors’ responsibilities were as follows – SMF, LMJ, LST: designed research; LMJ, LST: provided essential materials; SMF: analyzed data; SMF, LMJ, CLA, LSA, KM, DR, LST: wrote the paper; SMF: had primary responsibility for final content; and all authors: read and approved the final version.

## Conflict of interest

The authors report no conflicts of interest.

## Funding

This work was supported by Wellcome Trust. The funding source had no role in the study design; the collection, analysis, and interpretation of data; the writing of the report; or the decision to submit for publication.

## Data availability

Data described in the manuscript, code book, and analytic code will be made available upon request pending application and approval.
