# Supplementary material for: Adherence to the Planetary Health Diet Index and correlation with nutrients of public health concern: an analysis of NHANES 2003–2018
Source: Am J Clin Nutr. 2024 Jan 5;119(2):384–92. doi: 10.1016/j.ajcnut.2023.10.018 (PMC10884610; doi:10.1016/j.ajcnut.2023.10.018)
Supplement: Multimedia component 1 [file mmc1.docx]

**Adherence to the Planetary Health Diet Index and Correlation with Nutrients of Public Health Concern: An analysis of NHANES 2003-2018**

Sarah M. Frank, et al.

**Supplementary Methods**

Supplemental Table 1: Flattening of data for an example food reported in the National Health and Nutrition Examination Survey - Beef curry with rice^*^

**Supplemental Results**

Supplemental Figure 1: Flowchart of participant inclusion, National Health and Nutrition Examination Survey 2003-2018

Supplemental Table 2: Median Planetary Health Diet Index Score (95% CI) by Survey Cycle, National Health and Nutrition Examination Survey 2003-2018

Supplemental Table 3: Median intake of Planetary Health Diet Index components, National Health and Nutrition Examination Survey 2003-2018

Supplemental Table 4: Predicted probability of inadequate intake for key nutrients of concern by quintile of Planetary Health Diet Index, National Health and Nutrition Examination Survey 2003-2018


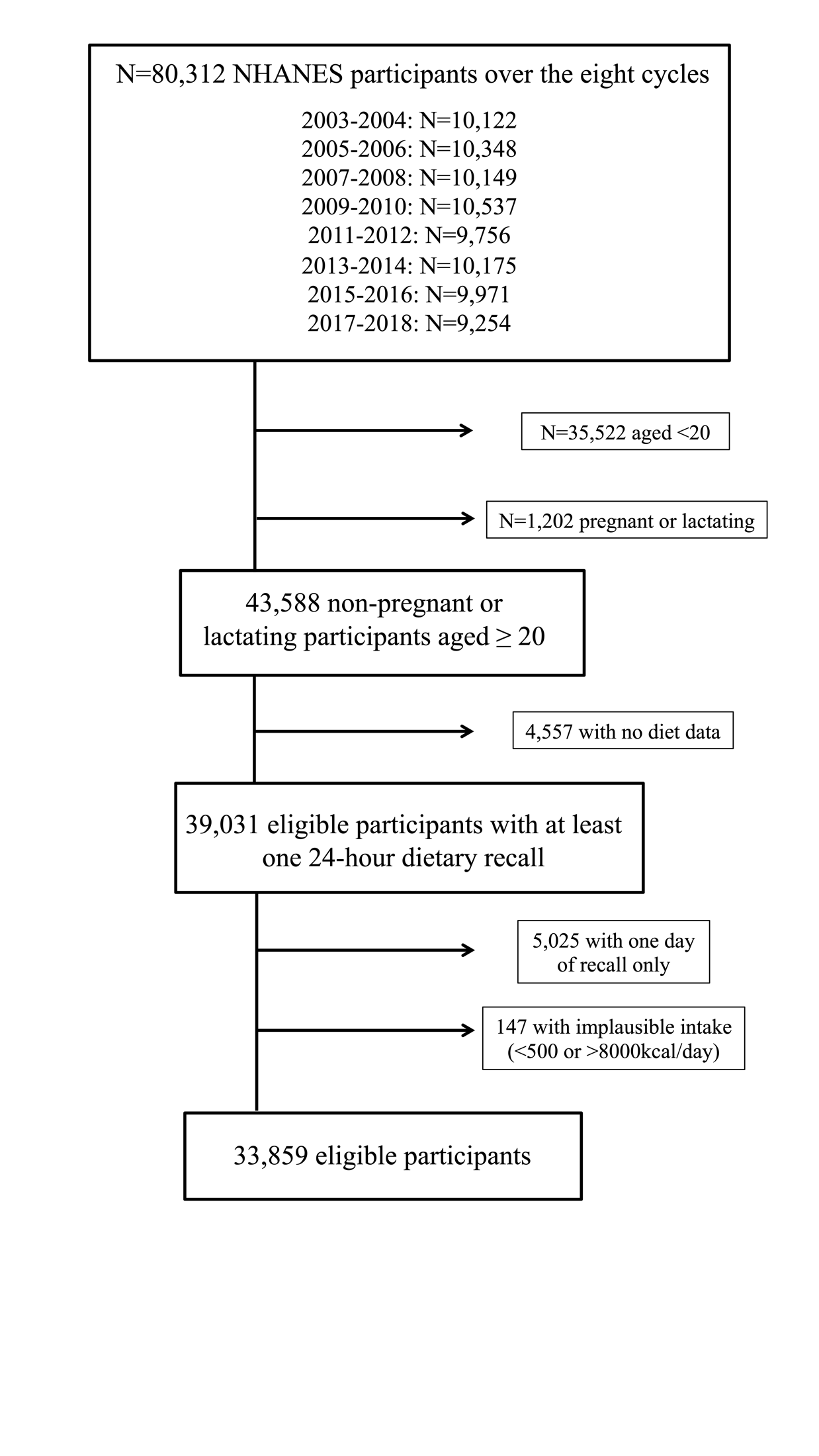


**Supplemental Figure 1:** Flowchart of participant inclusion, National Health and Nutrition Examination Survey 2003-2018

| **Supplemental Table 1:** Flattening of data for an example food reported in the National Health and Nutrition Examination Survey - Beef curry with rice^*^ | | | | |  |
| --- | --- | --- | --- | --- | --- |
| **Food code** | **Main Food Description** | **Ingredient code** | **Ingredient description** | **Ingredient weight per recipe**  **“Main Food Description”** | **Ingredient weight per recipe**  **“Beef curry with Rice”** |
| 27116110 | Beef curry with rice | 56205000 | Rice, cooked, NFS^†^ | 40g | 40g |
| 27116110 | Beef curry with rice | 27116100 | Beef curry | 60g | Carried forward to next step |
|  |  |  |  |  |  |
| 27116100 | Beef curry | 2047 | Salt, table, iodized | 0.5g | 0.3g |
| 27116100 | Beef curry | 23090 | Beef, chuck for stew, separable lean and fat, all grades, cooked, braised | 20g | 12g |
| 27116100 | Beef curry | 82101000 | Vegetable oil, NFS | 2g | 1.2g |
| 27116100 | Beef curry | 81312100 | Curry sauce | 40g | Carried forward to next step |
| 27116100 | Beef curry | 99997810 | Vegetables as ingredient in curry | 40g | Carried forward to next step |
|  |  |  |  |  |  |
| 99997810 | Vegetables as ingredient in curry | 11313 | Peas, green, frozen, cooked, boiled, drained, without salt | 5g | 1.2g |
| 99997810 | Vegetables as ingredient in curry | 11674 | Potatoes, baked, flesh and skin, without salt | 25g | 6.0g |
| 99997810 | Vegetables as ingredient in curry | 99997310 | Carrots, cooked, as ingredient | 10g | 2.4g |
| 99997810 | Vegetables as ingredient in curry | 99997410 | Tomatoes, cooked, as ingredient | 15g | 3.6g |
| 99997810 | Vegetables as ingredient in curry | 99997510 | Onions, cooked, as ingredient | 25g | 6.0g |
| 99997810 | Vegetables as ingredient in curry | 99997520 | Green pepper, cooked, as ingredient | 5g | 1.2g |
| 99997810 | Vegetables as ingredient in curry | 99997535 | Cauliflower, cooked, as ingredient | 10g | 2.4g |
| 99997810 | Vegetables as ingredient in curry | 99997545 | Green beans, cooked, as ingredient | 5g | 1.2g |
|  |  |  |  |  |  |
| 81312100 | Curry sauce | 1116 | Yogurt, plain, whole milk | 0.41 cup-equiv. | 0.1 cup-equiv |
| 81312100 | Curry sauce | 11549 | Tomato products, canned, sauce | 80g | 19.2g |
| 81312100 | Curry sauce | 12117 | Nuts, coconut milk, raw (liquid expressed from grated meat and water) | 7g | 1.7g |
| 81312100 | Curry sauce | 82101000 | Vegetable oil, NFS | 6g | 1.4g |

^*^ Complete code for flattening available at: <https://github.com/thrabchak/usda-nutrition-recipe-flattening>

^†^ Represents the amount of uncooked grains as detailed in the Food Patterns Equivalents Database Methodology.

| **Supplemental Table 2:** Median Planetary Health Diet Index Score (95% CI) by Survey Cycle, National Health and Nutrition Examination Survey 2003-2018^*,†^ | | | | | | | | |
| --- | --- | --- | --- | --- | --- | --- | --- | --- |
|  | 2003-2004 | 2005-2006 | 2007-2008 | 2009-2010 | 2011-2012 | 2013-2014 | 2015-2016 | 2017-2018 |
| Quintile 1 | 47.3 (46.6, 48.1) | 48.5 (47.6, 49.4) | 47.9 (47.0, 48.7) | 48.5 (47.5, 49.6) | 50.0^***^ (49.0, 50.9) | 49.3^**^ (48.3, 50.3) | 51.0^***^ (49.7, 52.2) | 51.5^***^ (50.4, 52.6) |
| Quintile 5 | 78.7 (77.7, 79.8) | 81.2^**^ (80.0, 82.3) | 81.6^***^ (80.4, 82.8) | 84.1^***^ (83.2, 85.0) | 86.4^***^ (85.3, 87.6) | 85.2^***^ (84.2, 86.2) | 87.3^***^ (86.0, 88.6) | 85.5^***^ (84.2, 86.8) |
| Overall | 62.7 (62.0, 63.4) | 64.0 (63.3, 64.6) | 63.3 (62.6, 64.0) | 64.9^***^ (64.2, 65.6) | 67.6^***^ (66.7, 68.5) | 67.6^***^ (66.6, 68.5) | 67.9^***^ (67.1, 68.7) | 66.9^***^ (66.2, 67.7) |
| ^*^ Quantile regression models were adjusted for total energy intake  ^†^ * p<0.05, ** p<0.01, *** p<0.001 for the difference from the 2003-2004 NHANES cycle | | | | | | | | |

| **Supplemental Table 3:** Median intake of Planetary Health Diet Index components, National Health and Nutrition Examination Survey 2003-2018^*,† ,‡^ | | | | | | | | | |
| --- | --- | --- | --- | --- | --- | --- | --- | --- | --- |
|  | 2003-2004 | 2005-2006 | 2007-2008 | 2009-2010 | 2011-2012 | 2013-2014 | 2015-2016 | 2017-2018 | Overall |
| *Encouraged components* | |  |  |  |  |  |  |  |  |
| Whole grain | 16.0 (13.6-18.4) | 23.6^***^(21.2-26.1) | 23.3^***^(20.9-25.7) | 27.6^***^(25.6-29.7) | 30.4^***^(27.0-33.7) | 27.3^***^(24.1-30.6) | 29.6^***^(26.0-33.1) | 23.9^***^(20.2-27.6) | 24.7 |
| Whole fruit (excludes fruit juice) | 57.4 (51.1-63.8) | 59.5 (53.2-65.8) | 62.1 (57.3-66.8) | 69.1^**^(63.8-74.3) | 63.8 (58.9-68.6) | 65.7 (60.0-71.3) | 59.3 (53.5-65.1) | 59.5 (51.4-67.6) | 61.7 |
| Non-starchy vegetables | 136.2 (130.1-142.2) | 134.2 (129.3-139.2) | 126.5^*^(120.6-132.3) | 131.0 (126.4-135.7) | 135.6 (129.6-141.5) | 126.5^*^(121.0-132.0) | 123.8^**^(118.0-129.7) | 118.7^***^(111.9-125.4) | 127.3 |
| Nuts and seeds | 1.3 (1.0-1.5) | 1.4 (1.2-1.7) | 1.3 (1.1-1.6) | 1.4 (1.1-1.7) | 1.9^**^(1.5-2.4) | 1.6 (1.2-2.0) | 2.2^**^(1.6-2.8) | 2.2^**^(1.5-3.0) | 2.1 |
| Non-soy legumes | 0.0 (0.0-0.0) | 0.0 (0.0-0.0) | 0.0 (0.0-0.0) | 0.0 (0.0-0.0) | 0.0 (0.0-0.0) | 0.0 (0.0-0.0) | 0.0 (0.0-0.0) | 0.0 (0.0-0.0) | 0 |
| Soy foods | 0.0 (0.0-0.0) | 0.0 (0.0-0.0) | 0.0 (0.0-0.0) | 0.0 (0.0-0.0) | 0.0 (0.0-0.0) | 0.0 (0.0-0.0) | 0.0 (0.0-0.0) | 0.0 (0.0-0.0) | 0 |
| Added fat - unsaturated oils^§^ | 6.1 (5.9-6.3) | 7.1^***^(6.8-7.3) | 7.2^***^(7.0-7.4) | 7.7^***^(7.5-7.9) | 8.8^***^(8.5-9.1) | 8.9^***^(8.6-9.1) | 9.8^***^(9.5-10.1) | 10.3^***^(10.0-10.6) | 7.9 |
| *Discouraged components* | |  |  |  |  |  |  |  |  |
| Starchy vegetables | 47.8 (44.4-51.2) | 45.6 (42.4-48.9) | 49.0 (46.0-52.0) | 44.4 (41.5-47.3) | 43.3 (41.0-45.6) | 39.6^***^(36.5-42.8) | 39.4^***^(35.9-42.9) | 39.0^***^(35.0-43.0) | 40.5 |
| Dairy foods^¶^ | 1.3 (1.3, 1.4) | 1.4^**^ (1.4, 1.5) | 1.4 (1.3, 1.4) | 1.5^***^ (1.4, 1.5) | 1.4^**^ (1.4, 1.5) | 1.4^**^ (1.4, 1.5) | 1.4 (1.3, 1.4) | 1.3 (1.2, 1.4) | 1.3 |
| Red/processed meat | 74.5 (70.7-78.3) | 76.2 (73.2-79.2) | 72.6 (69.1-76.0) | 70.9 (67.7-74.2) | 71.1 (67.6-74.7) | 70.2 (66.0-74.5) | 67.3 (64.1-70.5) | 67.2 (63.0-71.4) | 65.9 |
| Poultry | 23.1 (19.3-26.7) | 29.0 (25.8-32.1) | 32.6^***^(29.6-35.7) | 27.7 (24.2-31.2) | 27.9 (24.4-31.3) | 29.6^**^(26.9-32.4) | 29.6 (26.1-33.2) | 30.5^**^(26.7-34.3) | 27.7 |
| Eggs | 8.6 (7.6-9.7) | 12.1^***^(11.2-13.0) | 12.9^***^(11.8-14.0) | 11.9^***^(10.8-13.0) | 11.9^***^(10.5-13.3) | 13.6^***^(12.1-15.0) | 13.0^***^(11.4-14.7) | 13.1^***^(11.3-15.0) | 11.0 |
| Fish/shellfish | 0.0 (0.0-0.0) | 0.0 (0.0-0.0) | 0.0 (0.0-0.0) | 0.0 (0.0-0.0) | 0.0 (0.0-0.0) | 0.0 (0.0-0.0) | 0.0 (0.0-0.0) | 0.0 (0.0-0.0) | 0 |
| Added fat - saturated oils and *trans* fat^§^ | 9.8 (9.5-10.1) | 8.6^***^(8.3-8.8) | 8.2^***^(8.0-8.5) | 7.9^***^(7.6-8.2) | 7.1^***^(6.8-7.4) | 6.9^***^(6.6-7.2) | 7.4^***^(7.0-7.7) | 7.5^***^(7.2-7.8) | 8.3 |
| Added sugar and  fruit juice^§^ | 14.9 (14.4-15.4) | 14.0^**^(13.5-14.6) | 13.9^**^(13.4-14.4) | 13.3^***^(12.9-13.7) | 12.9^***^(12.4-13.4) | 12.6^***(^12.2-13.1) | 11.8^***^(11.3-12.3) | 11.9^***^(11.4-12.4) | 13.1 |
| ^*^ Quantile regression models were adjusted for total energy intake.  ^†^  Values are median (95% CI) grams of food group intake unless otherwise indicated  ^‡^ * p<0.05, ** p<0.01, *** p<0.001 for the difference from Quintile 1  ^§^ Values are percent (95% CI) of total energy intake  ^¶^ Values are cup-equivalents (95% CI) | | | | | | | | | |

| **Supplemental Table 4:** Predicted probability of inadequate intake for key nutrients of concern by quintile of Planetary Health Diet Index, National Health and Nutrition Examination Survey 2003-2018^*,†^ | | | | | |
| --- | --- | --- | --- | --- | --- |
|  | Quintile 1 | Quintile 2 | Quintile 3 | Quintile 4 | Quintile 5 |
| **Iron** | 4.3 (3.8, 4.7) | 4.8 (4.4, 5.2) | 4.7 (4.3, 5.2) | 3.6^*^ (3.1, 4.1) | 3.1^***^ (2.8, 3.3) |
| **Fiber** | 99.8 (99.7, 99.9) | 99.1^*^ (98.9, 99.3) | 97.2^**^ (96.8, 97.6) | 92.1^***^ (91.2, 93.0) | 73.7^***^ (71.4, 76.0) |
| **Potassium** | 76.1 (73.8, 78.3) | 75.0 (73.4, 76.6) | 70.3^**^ (68.2, 72.4) | 62.8^***^ (61.2, 64.5) | 51.0^***^ (48.5, 53.5) |
| **Calcium** | 37.1 (35.1, 39.2) | 44.5^***^ (42.7, 46.4) | 46.0^***^ (43.1, 48.9) | 45.5^***^ (43.5, 47.5) | 44.3^***^ (42.3, 46.3) |
| ^*^ Results are from the Simulating Intake of Micronutrients for Policy Learning and Engagement (SIMPLE) macro wrapper of the National Cancer Institute (NCI) Method for Estimating Usual Intake and were adjusted for age, sex, income, education, race/ethnicity, and total energy intake  ^†^  * p<0.05, ** p<0.01, *** p<0.001 for the difference from Quintile 1 | | | | | |
